# Bone and ocular safety of budesonide/glycopyrrolate/formoterol fumarate metered dose inhaler in COPD: a 52-week randomized study

**DOI:** 10.1186/s12931-019-1126-7

**Published:** 2019-07-29

**Authors:** Edward M. Kerwin, Gary T. Ferguson, Mindy Mo, Kiernan DeAngelis, Paul Dorinsky

**Affiliations:** 1 Clinical Research Institute of Southern Oregon, 3860 Crater Lake Avenue, Medford, OR 97504 USA; 2 Pulmonary Research Institute of Southeast Michigan, Farmington Hills, MI USA; 3 Formerly of AstraZeneca, Morristown, NJ USA; 4 AstraZeneca, Durham, NC USA

**Keywords:** BGF MDI, Bone mineral density, Inhaled corticosteroid, Lens opacity, LOCS III, Metered dose inhaler

## Abstract

**Background:**

Long-term use of inhaled corticosteroids (ICSs) has been associated with increased risk of bone and ocular comorbidities. We evaluated the effects of the triple fixed-dose combination budesonide/glycopyrrolate/formoterol fumarate metered dose inhaler (BGF MDI), formulated using co-suspension delivery technology, on bone mineral density (BMD) and ocular safety in patients with moderate-to-very severe chronic obstructive pulmonary disease (COPD).

**Methods:**

In this extension study, a subset of patients from the 24-week, phase III, randomized, double-blind KRONOS study (NCT02497001) continued treatment (BGF MDI 320/18/9.6 μg, budesonide/formoterol fumarate [BFF] MDI 320/9.6 μg or glycopyrrolate/formoterol fumarate [GFF] MDI 18/9.6 μg, as a non-steroidal comparator) for an additional 28 weeks. Primary endpoints were percentage change from baseline in lumbar spine BMD and change from baseline in lens opacities classification system III posterior subcapsular cataract (P) score, both at Week 52. Adverse events were also assessed.

**Results:**

In total, 456 patients were included in the safety population (53.1% male, mean age 62.8 years). Changes from baseline in lumbar spine BMD (least squares mean [LSM] range − 0.12 to 0.38%) and P score (LSM range 0.02–0.15) were small for all treatments. Both BGF MDI and BFF MDI were non-inferior to GFF MDI using margins of −2% (BMD) and 0.5 units (P score). The incidence of treatment-emergent adverse events (TEAEs)  was generally similar among groups. Rates of confirmed pneumonia were low overall (2.4%) and highest in the GFF MDI group (3.4%), followed by BGF MDI (2.1%) and BFF MDI (1.1%). There were no cumulative adverse effects of treatment over time as the incidence and types of TEAEs, were generally similar in the first 24 weeks of the study and after Week 24.

**Conclusions:**

In patients with COPD, both ICS-containing therapies were non-inferior to GFF MDI for the primary BMD and ophthalmological endpoints. Changes from baseline in all three treatment groups over 52 weeks were small and not clinically meaningful. All treatments were well tolerated with no new or unexpected safety findings.

**Trial registration:**

ClinicalTrials.gov NCT02536508. Registered 27 August 2015.

**Electronic supplementary material:**

The online version of this article (10.1186/s12931-019-1126-7) contains supplementary material, which is available to authorized users.

## Introduction

Treatment with long-acting bronchodilators is central to the management of chronic obstructive pulmonary disease (COPD) [1]. For patients receiving dual long-acting bronchodilator therapy who experience continued COPD exacerbations, the addition of an inhaled corticosteroid (ICS) is a recommended treatment option [1], and triple therapies have been shown to improve lung function and quality of life, and reduce exacerbations versus corresponding dual long-acting bronchodilator therapies [2, 3]. However, concerns remain regarding possible consequences of long-term ICS use, including decreased bone density and the development of cataracts. These are especially relevant for patients with COPD as the majority are older adults [4], and risk of bone loss and ocular comorbidities increases with age [5, 6].

Furthermore, the incidence of osteoporosis and glaucoma is increased in patients with COPD even compared with age-matched controls [7], which may result from a combination of factors including corticosteroid use (oral and/or inhaled), smoking, and physiological complications of COPD including hypoxia and systemic inflammation [8–10]. Studies of ICS use that have investigated the risk of bone- and ocular-related adverse effects have varied in their findings [11–19], potentially due to differences in study duration, patient population, ICS compound used, and dose. Therefore, it is important to evaluate the impact of novel ICS-containing therapies on the incidence of these potential adverse effects.

Budesonide/glycopyrrolate/formoterol fumarate metered dose inhaler (BGF MDI), an ICS/long-acting anti-muscarinic antagonist (LAMA)/long-acting β_2_-agonist (LABA) triple fixed-dose combination (FDC) formulated using co-suspension delivery technology, had benefits on lung function, symptoms and exacerbations versus dual therapies in a pivotal 24-week, phase III study in patients with moderate-to-very severe COPD (KRONOS) [2]. To further assess the tolerability of BGF MDI over a longer treatment period, a subset of patients who participated in KRONOS continued into an extension study to examine the effects of triple therapy with BGF MDI and the corresponding dual therapies budesonide/formoterol fumarate (BFF) MDI and glycopyrrolate/formoterol fumarate (GFF) MDI on bone mineral density (BMD) and ocular safety over 52 weeks.

## Methods

### Study design and treatment

This was an extension study (registered with ClinicalTrials.gov; NCT02536508) conducted in a subset of patients from US sites who were initially enrolled in the 24-week KRONOS study (NCT02497001) and continued their randomized treatment for 28 additional weeks (Additional file 1: Figure S1). The KRONOS study was a randomized, double-blind, parallel-group, multicenter, phase III study in which patients with moderate-to-very severe COPD were randomized 2:2:1:1 to BGF MDI 320/18/9.6 μg, GFF MDI 18/9.6 μg, BFF MDI 320/9.6 μg, or open-label budesonide/formoterol fumarate dihydrate dry powder inhaler (DPI) 400/12 μg (Symbicort® Turbuhaler®) as an active control (all via two oral inhalations, twice daily) [2]. Details of the randomization have been described [2]. Glycopyrrolate 18 μg and formoterol fumarate 9.6 μg are equivalent to 14.4 μg of glycopyrronium and 10 μg of formoterol fumarate dihydrate. Patients randomized to budesonide/formoterol DPI in KRONOS were not enrolled in the extension study.

The entire 52-week study took place from 24 September 2015 to 6 September 2017. The informed consent form and study protocol were approved by an institutional review board (Schulman Associates; approval number 201503097), and patients provided written informed consent for both KRONOS and the extension study prior to screening. The study was conducted in accordance with the Declaration of Helsinki and the International Conference on Harmonisation/Good Clinical Practice, as well as applicable regulatory requirements.

### Study population

The inclusion/exclusion criteria for KRONOS [2] also applied to the extension study. Briefly, patients were 40–80 years of age with an established clinical history of COPD, a smoking history of ≥10 pack-years and a post-bronchodilator forced expiratory volume in 1 s (FEV_1_)/forced vital capacity ratio <0.70 and post-bronchodilator FEV_1_ <80% and ≥25% predicted normal value. Exclusion criteria specific to the 52-week study comprised severe osteoporosis, a T-score <−2.5 at baseline or inability to achieve an acceptable BMD scan (BMD exclusion criteria); and inability to dilate pupil ≥6 mm, intraocular pressure (IOP) ≥21 mmHg (lowest of 3 readings), or an implanted artificial intraocular lens (ophthalmological exclusion criteria). Important protocol amendments are shown in Additional file 1: Table S1.

### Bone and ocular safety endpoints

BMD endpoints included the percentage change from baseline in BMD of lumbar spine segments 2–4 (L2–L4) at Week 52 (primary BMD endpoint) and percentage change from baseline in BMD of the total hip at Week 52 (other BMD endpoint). BMD was evaluated at baseline (Day 1 of the KRONOS study, prior to randomization) and Week 52 using dual-energy x-ray absorptiometry, with two scans taken at each site.

The primary ophthalmological endpoint was the change from baseline (assessed during screening in KRONOS) in the lens opacities classification system III (LOCS III) posterior subcapsular cataract (P) score at Week 52. Other ophthalmological endpoints (all at Week 52) included: changes from baseline in LOCS III cortical cataract (C), nuclear color (NC), and nuclear opalescence (NO) scores; changes from baseline in IOP, logarithm of the minimum angle of resolution (LogMAR) visual acuity scores, and horizontal cup-to-disc ratio; the proportion of patients with LOCS III grade increases of ≥0.5, ≥1.0, or ≥ 1.5 units in each of the 4 scales; and the proportion of patients with IOP ≥22 mmHg or change from baseline in IOP of ≥7 mmHg. Further details regarding the bone and ocular safety assessments are provided in Additional file 1: supplementary methods.

### Additional safety evaluations

Safety was additionally assessed by adverse event (AE) monitoring, 12-lead electrocardiography, clinical laboratory testing, and vital sign measurements. Adverse events of special interest were identified based on the pharmacologically predictable effects of ICSs, LAMAs, and LABAs (further details are provided in Additional file 1: supplementary methods). Cases of pneumonia and major adverse cardiovascular events (MACE) were reviewed and adjudicated by an external, independent Clinical Endpoint Committee against predefined criteria.

### Efficacy endpoints

Efficacy endpoints assessed over 52 weeks included the change from baseline in average daily rescue salbutamol use, percentage of days with no rescue salbutamol use, rate of moderate/severe COPD exacerbations, and change from baseline in Exacerbations of Chronic Pulmonary Disease Tool (EXACT) and EXACT-Respiratory Symptom (RS) total scores. Patients continued to use an electronic diary provided during KRONOS to record study medication use, the total daily number of ‘puffs’ of rescue medication, COPD symptoms, and responses for EXACT (a 14-item questionnaire assessing patient-reported outcomes [20]).

### Statistical analysis

The safety and modified intent-to-treat populations included all patients who signed the informed consent form for the extension study and received any amount of study drug (other than budesonide/formoterol DPI), excluding those who did not meet eligibility criteria at baseline or had no data collected after the Week 24 transition visit. The extension study safety population was defined as all patients in the safety population who met the continued eligibility criteria for the extension study at the Week 24 transition visit or attended at least one visit during Weeks 28–52 (Additional file 1: Figure S1). The BMD and ophthalmological populations were defined as all evaluable patients in the safety population who had baseline and ≥1 on-treatment BMD or ophthalmological assessments, respectively, analyzed according to actual treatment received.

For the primary BMD endpoint, non-inferiority was declared if the lower 95% confidence interval (CI) bound for the percentage treatment difference in lumbar spine BMD was >−2%. Non-inferiority for the primary ophthalmological endpoint was declared if the upper 95% CI bound for the treatment difference in LOCS III P scores was <0.5 units. Because the primary objectives were related to safety, all hypotheses were tested at nominal alphas, and there were no controls for multiplicity. Further details are provided in Additional file 1: supplementary methods.

## Results

### Study population

**Fig. 1:**
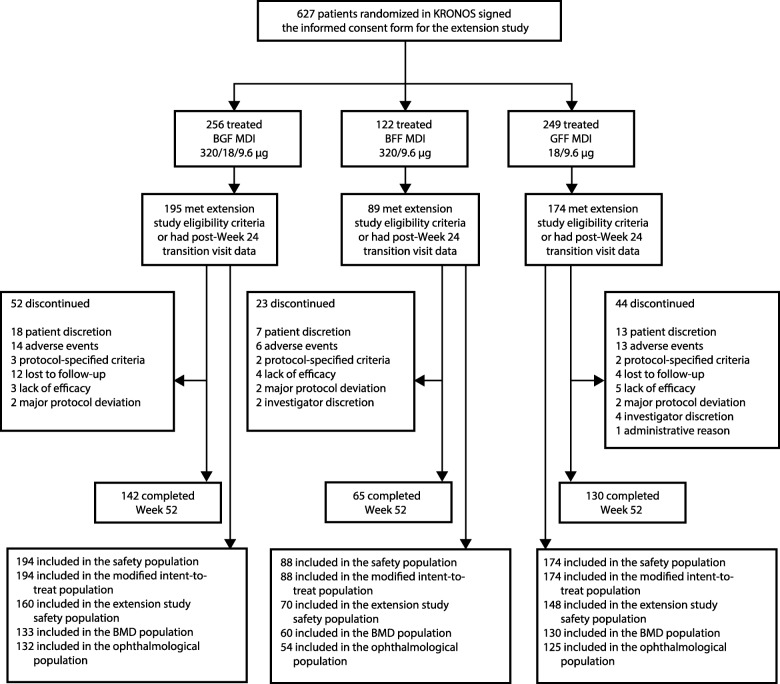
Patient disposition. Two patients (one in the BGF MDI group and one in the BFF MDI group) participated in multiple sponsor-led studies and were excluded from all analysis populations. *BFF* Budesonide/formoterol fumarate, *BGF* Budesonide/glycopyrrolate/formoterol fumarate, *BMD* Bone mineral density, *GFF* Glycopyrrolate/formoterol fumarate, *MDI* Metered dose inhaler

In total, 627 patients who were randomized in KRONOS consented to participate in the extension study (Fig. 1). Of these, 169 patients (27.0%) did not meet the extension study entry criteria at baseline and lacked post-Week 24 transition visit data (including 81 patients [12.9%] who had BMD T-scores <−2.5 at baseline), and were excluded from the study analysis populations. Seventy-two patients who had consented to participate in the extension study when they entered KRONOS discontinued treatment prior to Week 24. A quarter of these patients (*n* = 18) discontinued due to AEs, none of which were bone- or ocular-related (further details are provided in Additional file 1: supplementary results). The safety population included 456 patients (53.1% male, mean age of 62.8 years; Table 1). Demographic characteristics were generally similar across treatment groups. Mean study drug exposure was comparable across groups, ranging from 298.2 days (BFF MDI) to 310.4 days (GFF MDI). The percentage of patients with exposure ≥24 weeks (range 81.8 to 86.2%) and ≥48 weeks (range 72.2 to 75.0%) was similar across the treatment groups.

**Table 1:** Baseline demographics and clinical characteristics (safety population)

	BGF MDI 320/18/9.6 μg (*N* = 194)	BFF MDI 320/9.6 μg (*N* = 88)	GFF MDI 18/9.6 μg (*N* = 174)
Age, years (mean [SD])	62.6 (7.9)	64.0 (7.2)	62.4 (7.8)
Male, n (%)	102 (52.6)	53 (60.2)	87 (50.0)
Race, n (%)			
White	179 (92.3)	79 (89.8)	156 (89.7)
Black	13 (6.7)	9 (10.2)	17 (9.8)
Other	2 (1.0)	0	1 (0.6)
Ethnicity, *n* (%)			
Hispanic or Latino	9 (4.6)	6 (6.8)	5 (2.9)
Not Hispanic or Latino	185 (95.4)	82 (93.2)	169 (97.1)
Body mass index, kg·m^−2^ (mean [SD])	29.0 (7.4)	29.0 (5.8)	29.0 (6.5)
Current smoker, *n* (%)	101 (52.1)	42 (47.7)	95 (54.6)
Number of pack-years smoked^#^ (median [range])	45.0 (11.2–256.0)	47.3 (14.3–134.0)	50.0 (10.0–171.0)
COPD severity, *n* (%)			
Moderate	95 (49.0)	45 (51.1)	91 (52.3)
Severe	86 (44.3)	37 (42.0)	65 (37.4)
Very severe	13 (6.7)	6 (6.8)	18 (10.3)
COPD duration, years (mean [SD])	8.6 (6.7)	9.6 (6.3)	7.7 (5.3)
Moderate/severe COPD exacerbations in the past 12 months, *n* (%)			
0	152 (78.4)	67 (76.1)	129 (74.1)
1	33 (17.0)	18 (20.5)	34 (19.5)
≥2	9 (4.6)	3 (3.4)	11 (6.3)
Eosinophil count, cells·mm^− 3^ (median, [range])	180 (10–655)	190 (15–505)	185 (40–2490)
<150 cells**·**mm^− 3^, *n* (%)	68 (35.1)	32 (36.4)	54 (31.0)
≥150 cells**·**mm^−3^, *n* (%)	126 (64.9)	56 (63.6)	120 (69.0)
Use of ICS at screening, *n* (%)	152 (78.4)	73 (83.0)	127 (73.0)
CAT total score (mean [SD])	21.2 (6.4)*n* = 192	22.3 (6.7) *n* = 86	20.4 (6.3) *n* = 172
EXACT total score (mean [SD])^¶^	35.2 (10.8)*n* = 194	35.7 (10.4) *n* = 86	34.6 (10.6) *n* = 174
Rescue medication use, puffs·day^− 1^ (median [range])^¶^	1.9 (0.0–12.0)*n* = 194	2.4 (0.0–17.7) *n* = 86	2.0 (0.0–18.2) *n* = 174

*BFF* Budesonide/formoterol fumarate, *BGF* Budesonide/glycopyrrolate/formoterol fumarate, *CAT* COPD Assessment Test, *COPD* Chronic obstructive pulmonary disease, *EXACT* Exacerbations of Chronic Pulmonary Disease Tool, *GFF* Glycopyrrolate/formoterol fumarate, *ICS* Inhaled corticosteroid, *MDI* Metered dose inhaler, *mITT* Modified intent-to-treat, *SD* Standard deviation. ^#^: number of pack-years smoked = (number of cigarettes each day/20) × number of years smoked, ^¶^: mITT population

### BMD endpoints

Baseline BMD characteristics were comparable across treatment groups (Table 2), and the majority of patients had normal BMD (T-scores >−1) at the lumbar spine (72.0%) and hip (59.9%). The mean T-scores in the BMD population at baseline were 0.1 for lumbar spine BMD and −0.6 for hip BMD, which were comparable to those in patients who withdrew from the study prior to Week 24 (0.0 and −0.7, respectively).

**Table 2:** Primary and other BMD endpoints (BMD population)

	BGF MDI 320/18/9.6 μg (*N* = 133)	BFF MDI 320/9.6 μg(*N* = 60)	GFF MDI 18/9.6 μg (*N* = 130)
Lumbar spine BMD (L2-L4)			
*n*	128	57	123
Baseline, g·cm^−2^ (mean [SD])	1.18 (0.21)	1.19 (0.20)	1.16 (0.18)
Change from baseline at Week 52, % (primary BMD endpoint)			
LSM	−0.09	−0.12	0.38
95% CI	(−0.74, 0.56)	(− 1.09, 0.86)	(−0.28, 1.04)
LSM % difference between treatments			
Versus GFF MDI 18/9.6 μg^#^			
LSM	−0.47	−0.50	NA
95% CI	(−1.38, 0.45)	(− 1.69, 0.68)	
Versus BFF MDI 320/9.6 μg^*¶*^			
LSM	0.03	NA	Shown above
95% CI	(−1.13, 1.20)		
Total hip BMD			
*n*	128	57	119
Baseline, g·cm^−2^(mean [SD])	0.94 (0.15)	0.98 (0.16)	0.93 (0.14)
Change from baseline at Week 52, %			
LSM	−0.87	−1.12	− 0.32
95% CI	(−1.39, −0.34)	(−1.90, − 0.33)	(− 0.86, 0.23)
LSM % difference between treatments^¶^			
Versus GFF MDI 18/9.6 μg			
LSM	−0.55	−0.81	NA
95% CI	(−1.30, 0.21)	(−1.79, 0.16)	
Versus BFF MDI 320/9.6 μg			
LSM	0.25	NA	Shown above
95% CI	(−0.70, 1.21)		

*BMD* Bone mineral density, B*FF* Budesonide/formoterol fumarate, *BGF* Budesonide/glycopyrrolate/formoterol fumarate, *CI* Confidence interval, *GFF* Glycopyrrolate/formoterol fumarate, *L* Lumbar spine segment, *LSM* Least squares mean, *MDI* Metered dose inhaler, *NA* Not applicable, *SD* Standard deviation, ^#^ non-inferiority was declared if the lower confidence bound for the percentage treatment difference was >−2%, ^*¶*^ not a pre-specified non-inferiority comparison

For the primary BMD endpoint, percentage changes from baseline at Week 52 were small (least squares mean [LSM] range −0.12 to 0.38%) and similar across treatment groups (Table 2; Additional file 1: Figure S2) and both ICS-containing treatments were non-inferior to GFF MDI. The percentage changes from baseline in total hip BMD (other BMD endpoint) were also small (LSM range −1.12 to −0.32%) and comparable for all three treatments (Table 2, Additional file 1: Figure S2).

T-scores were calculated at baseline and Week 52 to assess shifts in BMD status during the treatment period (Table 3). Lumbar spine T-scores worsened from normal (>−1) to osteopenic (>−2.5 and ≤−1) for 3.1, 5.3, and 3.3% of patients treated with BGF MDI, BFF MDI, and GFF MDI, respectively (Table 3). No patients shifted to a lumbar spine BMD T-score of ≤−2.5 (indicative of osteoporosis). Improvements in lumbar spine T-scores were observed for 1.6, 3.5, and 7.3% of patients treated with BGF MDI, BFF MDI, and GFF MDI, respectively. For total hip BMD, 2.3% (BGF MDI), 1.8% (BFF MDI), and 0.8% (GFF MDI) of patients shifted from an osteopenic to osteoporotic T-score; and 3.9, 8.8, and 3.4% of patients, respectively, shifted from normal to osteopenic (Table 3). In the BGF MDI, BFF MDI, and GFF MDI groups, 3.1, 1.8, and 0.8% of patients, respectively, improved from osteopenic at baseline to a normal T-score at Week 52.

**Table 3:** Shifts in BMD T-scores^#^ (baseline to Week 52; BMD population)

	*Baseline T-score*	*Post-baseline T-score*		
		≤−2.5	>−2.5 and ≤−1	>−1
		Osteoporotic	Osteopenic	Normal
Shifts in T-scores for lumbar spine BMD (L2–L4)				
BGF MDI 320/18/9.6 μg (*N* = 128)^*¶*^	≤−2.5 (*n* = 0)	0	0	0
	>−2.5 and ≤ −1 (*n* = 37)	0	35 (94.6)	2 (5.4)
	>−1 (*n* = 91)	0	4 (4.4)	87 (95.6)
BFF MDI 320/9.6 μg (*N* = 57)^*¶*^	≤−2.5 (*n* = 0)	0	0	0
	>−2.5 and ≤−1 (*n* = 16)	0	14 (87.5)	2 (12.5)
	>−1 (*n* = 41)	0	3 (7.3)	38 (92.7)
GFF MDI 18/9.6 μg (*N* = 123)^*¶*^	≤−2.5 (*n* = 0)	0	0	0
	>−2.5 and ≤−1 (*n* = 35)	0	26 (74.3)	9 (25.7)
	>−1 (*n* = 88)	0	4 (4.5)	84 (95.5)
Shifts in T-scores for total hip BMD				
BGF MDI 320/18/9.6 μg (*N* = 128)^*¶*^	≤−2.5 (*n* = 0)	0	0	0
	>−2.5 and ≤−1 (*n* = 52)	3 (5.8)	45 (86.5)	4 (7.7)
	>−1 (*n* = 76)	0	5 (6.6)	71 (93.4)
BFF MDI 320/9.6 μg (*N* = 57)^*¶*^	≤−2.5 (*n* = 0)	0	0	0
	>−2.5 and ≤−1 (*n* = 18)	1 (5.6)	16 (88.9)	1 (5.6)
	>− 1 (*n* = 39)	0	5 (12.8)	34 (87.2)
GFF MDI 18/9.6 μg (*N* = 119)^*¶*^	≤−2.5 (*n* = 0)	0	0	0
	>−2.5 and ≤−1 (*n* = 52)	1 (1.9)	50 (96.2)	1 (1.9)
	>−1 (*n* = 67)	0	4 (6.0)	63 (94.0)

Data are n (%). *BMD* Bone mineral density, *BFF* Budesonide/formoterol fumarate, *BGF* Budesonide/glycopyrrolate/formoterol fumarate, *GFF* Glycopyrrolate/formoterol fumarate, *L* Lumbar spine segment, *MDI* Metered dose inhaler. ^#^: shifts are from baseline to the worst post-baseline value, ^*¶*^: *N* = number of patients with available data for both baseline and post-baseline T-scores (non-missing)

### Ophthalmological endpoints

At baseline, most patients (>94% overall) had normal horizontal cup-to-disc ratio (mean 0.3, range 0.0–1.0) and IOP (mean 14.9, range 8.0–25.0) and were not taking ophthalmological medications as eye drops. Baseline ophthalmological characteristics were similar across treatment groups (Table 4), although the GFF MDI group had slightly lower LOCS III scores at baseline compared to the BGF MDI group. Changes from baseline in LOCS III (P) score at Week 52 (primary ophthalmological endpoint) were small (LSM range 0.02–0.15) and similar among treatment groups (Table 4, Additional file 1: Figure S3); BGF MDI was non-inferior to GFF MDI. Similarly, changes from baseline in LOCS III NO, NC, and C scores at Week 52 were small (LSM ≤0.26), with the upper bound of the 95% CI consistently below the clinically meaningful value of 0.5 for BGF MDI versus GFF MDI (Table 4, Additional file 1: Figure S3). Changes from baseline in IOP at Week 52 were small and similar in the BGF MDI (0.67 mmHg) and GFF MDI (0.68 mmHg) treatment groups (Table 4), below the clinically meaningful value of 2 mmHg. There were no changes from baseline (in both eyes) in LogMAR visual acuity or horizontal cup-to-disc ratio across the treatment groups at Week 52 (not shown).

**Table 4:** Primary and other ophthalmological endpoints^#^ (ophthalmological population)

	BGF MDI 320/18/9.6 μg (*N* = 132)	BFF MDI 320/9.6 μg (*N* = 54)	GFF MDI 18/9.6 μg (*N* = 125)
LOCS III P score			
*n*	218	98	184
Baseline (mean [SD])	0.381 (0.880)	0.397 (0.650)	0.308 (0.567)
Change from baseline to Week 52 (primary ophthalmological endpoint)			
LSM	0.153	0.022	0.026
95% CI	(0.079, 0.227)	(−0.090, 0.135)	(−0.055, 0.106)
LSM difference between treatments			
Versus GFF MDI 18/9.6 μg^¶^			
LSM	0.127	−0.003	NA
95% CI	(0.017, 0.237)	(− 0.142, 0.135)	
Versus BFF MDI 320/9.6 μg^§^			
LSM	0.130	NA	Shown above
95% CI	(−0.004, 0.265)		
LOCS III NO score			
*n*	220	98	187
Baseline (mean [SD])	2.447 (1.082)	2.336 (0.886)	2.309 (1.060)
Change from baseline to Week 52			
LSM	0.255 (0.170, 0.340)	0.186 (0.059, 0.314)	0.047 (−0.045, 0.138)
95% CI			
LSM difference between treatments^§^			
Versus GFF MDI 18/9.6 μg			
LSM	0.208	0.140	NA
95% CI	(0.084, 0.333)	(−0.017, 0.297)	
Versus BFF MDI 320/9.6 μg			
LSM	0.069	NA	Shown above
95% CI	(−0.084, 0.222)		
LOCS III NC score			
*n*	219	98	187
Baseline (mean [SD])	2.290 (1.137)	2.287 (0.951)	2.178 (0.858)
Change from baseline to Week 52			
LSM	0.130	0.142	0.163
95% CI	(0.050, 0.209)	(0.022, 0.263)	(0.077, 0.248)
LSM difference between treatments^§^			
Versus GFF MDI 18/9.6 μg			
LSM	−0.033	−0.021	NA
95% CI	(−0.150, 0.084)	(−0.169, 0.128)	
Versus BFF MDI 320/9.6 μg			
LSM	−0.013	NA	Shown above
95% CI	(−0.157, 0.132)		
LOCS III C score			
*n*	218	98	187
Baseline (mean [SD])	0.832 (1.189)	0.801 (0.976)	0.727 (1.060)
Change from baseline to Week 52			
LSM	0.105 (0.022, 0.187)	0.170 (0.047, 0.294)	0.067 (−0.022, 0.155)
95% CI			
LSM difference between treatments^§^			
Versus GFF MDI 18/9.6 μg			
LSM	0.038	0.103	NA
95% CI	(−0.083, 0.159)	(−0.049, 0.256)	
Versus BFF MDI 320/9.6 μg			
LSM	−0.065	NA	Shown above
95% CI	(−0.214, 0.083)		
IOP, mmHg			
*n*	228	100	203
Baseline (mean [SD])	14.482 (2.908)	15.490 (2.740)	14.978 (2.971)
Change from baseline to Week 52			
LSM	0.670	0.178	0.680
95% CI	(0.330, 1.010)	(−0.339, 0.696)	(0.321, 1.039)
LSM difference between treatments^§^			
Versus GFF MDI 18/9.6 μg			
LSM	−0.010	−0.502	NA
95% CI	(−0.505, 0.486)	(−1.131, 0.128)	
Versus BFF MDI 320/9.6 μg			
LSM	0.492	NA	Shown above
95% CI	(−0.129, 1.113)		

*BFF* Budesonide/formoterol fumarate, *BGF* Budesonide/glycopyrrolate/formoterol fumarate, *C* Cortical cataract, *CI* Confidence interval, *GFF* Glycopyrrolate/formoterol fumarate, *IOP* Intraocular pressure, *LOCS III* Lens opacities classification system III, *LSM* Least squares mean, *MDI* Metered dose inhaler, *NA* Not applicable, *NC* Nuclear color, *NO* Nuclear opalescence, *P* Posterior subcapsular cataract, *SD* Standard deviation. ^#^: data presented are across eyes (irrespective of person) such that *n* = total number of eyes assessed, ^*¶*^: non-inferiority was declared if the upper confidence bound for the treatment difference was <0.5, ^§^: not a pre-specified non-inferiority comparison

Both ICS-containing treatment groups (BGF MDI and BFF MDI) had numerically greater proportions of patients relative to GFF MDI with Class 1 (≥0.5) increases in P score, and Class 2 (≥1.0) increases in C score (Table 5). BGF MDI also had numerically greater proportions of patients relative to BFF MDI and GFF MDI with Class 2 increases in NO score and P score. A numerically greater proportion of patients receiving GFF MDI had Class 1 and 2 increases in NC scores relative to BGF MDI and BFF MDI. Importantly, few patients (≤8.2%) across treatment groups had a grade increase of ≥1.5 (Class 3) in LOCS III scores of any scale at Week 52 (Table 5). Finally, few patients had an IOP ≥22 mmHg or an increase from baseline ≥7 mmHg in either eye, with similar proportions across the treatment groups (Table 5).

**Table 5:** Shifts in LOCS III scores and IOP (Week 52; ophthalmological population)

	BGF MDI 320/18/9.6 μg (*N* = 132)	BFF MDI 320/9.6 μg (*N* = 54)	GFF MDI 18/9.6 μg (*N* = 125)
Proportion of patients with increases in LOCS III P score, *n* (%)			
*n*	111	49	94
Class 1 (≥0.5 unit)	16 (14.4)	6 (12.2)	7 (7.4)
Class 2 (≥1.0 unit)	12 (10.8)	3 (6.1)	5 (5.3)
Class 3 (≥1.5 units)	8 (7.2)	0	4 (4.3)
Proportion of patients with increases in LOCS III NO score, *n* (%)			
*n*	111	49	95
Class 1 (≥0.5 unit)	40 (36.0)	16 (32.7)	33 (34.7)
Class 2 (≥1.0 unit)	19 (17.1)	5 (10.2)	11 (11.6)
Class 3 (≥1.5 units)	6 (5.4)	2 (4.1)	3 (3.2)
Proportion of patients with increases in LOCS III NC score, *n* (%)			
*n*	111	49	95
Class 1 (≥0.5 unit)	30 (27.0)	12 (24.5)	32 (33.7)
Class 2 (≥1.0 unit)	11 (9.9)	4 (8.2)	14 (14.7)
Class 3 (≥1.5 units)	5 (4.5)	1 (2.0)	3 (3.2)
Proportion of patients with increases in LOCS III C score, *n* (%)			
*n*	110	49	95
Class 1 (≥0.5 unit)	25 (22.7)	10 (20.4)	17 (17.9)
Class 2 (≥1.0 unit)	15 (13.6)	6 (12.2)	5 (5.3)
Class 3 (≥1.5 units)	8 (7.3)	4 (8.2)	4 (4.2)
Proportion of patients with IOP ≥22 mmHg or increase in IOP of ≥7 mmHg, *n* (%)^#^			
*n*	114	50	103
≥22 mmHg	2 (1.8)	3 (6.0)	4 (3.9)
≥7 mmHg increase	5 (4.4)	2 (4.0)	3 (2.9)

*BFF* Budesonide/formoterol fumarate, *BGF* Budesonide/glycopyrrolate/formoterol fumarate, *C* Cortical cataract, *GFF* Glycopyrrolate/formoterol fumarate, *IOP* Intraocular pressure, *LOCS III* Lens opacities classification system III, *MDI* Metered dose inhaler, *NC* Nuclear color, *NO* Nuclear opalescence, *P* Posterior subcapsular cataract. ^#^: in either eye

### Adverse events

Overall, the majority of patients experienced at least one treatment-emergent AE (TEAE); most were mild or moderate in intensity and the majority were considered not treatment-related by the investigator (Table 6). The most commonly reported TEAEs overall were upper respiratory tract infection, bronchitis, COPD (recorded as a TEAE only if meeting the criteria for a serious TEAE), and urinary tract infection (Table 6). In general, the incidence and types of TEAEs were generally similar across treatment groups. COPD (*n* = 22; 4.8%) and pneumonia (*n* = 6; 1.3%) were the most frequently reported serious TEAEs; all others occurred in ≤2 patients (0.4%) overall. The incidence of AEs of special interest, including bone- and ocular-related AEs, was low and similar across treatment groups. Overall, 61.1% (57.1–68.8% across treatment groups) and 55.6% (51.4–58.1% across treatment groups) of patients experienced TEAEs in the first 24 weeks and after Week 24 of the study, respectively (Additional file 1: Table S2). The pattern and frequency of individual TEAEs were generally similar across treatment groups during the first 24 weeks and after Week 24. During the first 24 weeks of the study, the most commonly reported TEAEs overall were upper respiratory tract infection, nasopharyngitis, and muscle spasms; after Week 24, the most commonly reported were upper respiratory tract infection, viral upper respiratory tract infection, and COPD (Additional file 1: Table S2).

**Table 6:** Summary of TEAEs (safety population)

TEAEs, *n* (%)	BGF MDI 320/18/9.6 μg (*N* = 194)	BFF MDI 320/9.6 μg (*N* = 88)	GFF MDI 18/9.6 μg (*N* = 174)	All patients (*N* = 456)
Patients with ≥1 TEAE	144 (74.2)	64 (72.7)	133 (76.4)	341 (74.8)
Mild	50 (25.8)	27 (30.7)	46 (26.4)	123 (27.0)
Moderate	63 (32.5)	29 (33.0)	65 (37.4)	157 (34.4)
Severe	31 (16.0)	8 (9.1)	22 (12.6)	61 (13.4)
Patients with treatment-related TEAEs^#^	35 (18.0)	17 (19.3)	29 (16.7)	81 (17.8)
Patients with serious TEAEs^§^	33 (17.0)	7 (8.0)	22 (12.6)	62 (13.6)
COPD^+^	12 (6.2)	1 (1.1)	9 (5.2)	22 (4.8)
Pneumonia	2 (1.0)	0	4 (2.3)	6 (1.3)
Patients with treatment-related serious TEAEs^#,§^	2 (1.0)	0	2 (1.1)	4 (0.9)
Patients with TEAEs leading to early discontinuation	16 (8.2)	6 (6.8)	12 (6.9)	34 (7.5)
Patients with confirmed MACE^¶^	3 (1.5)	0	3 (1.7)	6 (1.3)
Patients with confirmed pneumonia^¶^	4 (2.1)	1 (1.1)	6 (3.4)	11 (2.4)
Deaths (all causes)	3 (1.5)	0	1 (0.6)	4 (0.9)
TEAEs occurring in ≥4% of patients in any treatment arm				
Upper respiratory tract infection	18 (9.3)	6 (6.8)	18 (10.3)	42 (9.2)
Bronchitis	12 (6.2)	2 (2.3)	8 (4.6)	22 (4.8)
COPD^+^	12 (6.2)	1 (1.1)	9 (5.2)	22 (4.8)
Urinary tract infection	10 (5.2)	5 (5.7)	6 (3.4)	21 (4.6)
Muscle spasms	6 (3.1)	9 (10.2)	5 (2.9)	20 (4.4)
Viral upper respiratory tract infection	9 (4.6)	6 (6.8)	5 (2.9)	20 (4.4)
Sinusitis	11 (5.7)	2 (2.3)	6 (3.4)	19 (4.2)
Hypertension	8 (4.1)	4 (4.5)	6 (3.4)	18 (3.9)
Back pain	9 (4.6)	1 (1.1)	7 (4.0)	17 (3.7)
Nasopharyngitis	7 (3.6)	4 (4.5)	6 (3.4)	17 (3.7)
Diarrhea	5 (2.6)	2 (2.3)	9 (5.2)	16 (3.5)
Dyspnea	4 (2.1)	5 (5.7)	5 (2.9)	14 (3.1)
Pneumonia	5 (2.6)	1 (1.1)	8 (4.6)	14 (3.1)
Dysphonia	6 (3.1)	5 (5.7)	2 (1.1)	13 (2.9)
Bone- and ocular-related TEAEs occurring in ≥1% of patients				
Cataract	6 (3.1)	2 (2.3)	0	8 (1.8)
IOP increased	2 (1.0)	1 (1.1)	4 (2.3)	7 (1.5)
Osteoarthritis	2 (1.0)	0	4 (2.3)	6 (1.3)
Osteoporosis	2 (1.0)	1 (1.1)	2 (1.1)	5 (1.1)

*BFF* Budesonide/formoterol fumarate, *BGF* Budesonide/glycopyrrolate/formoterol fumarate, *COPD* Chronic obstructive pulmonary disease, *GFF* Glycopyrrolate/formoterol fumarate, *IOP* Intraocular pressure, *MACE* Major adverse cardiovascular event, *MDI* Metered dose inhaler, *TEAE* Treatment-emergent adverse event. ^#^: possibly, probably, or definitely related in the opinion of the investigator, ^¶^: confirmed by clinical endpoint committee, ^§^: TEAEs were classified as serious in the opinion of the investigator if they resulted in hospitalization or substantial disruption of the ability to conduct normal life functions, or were life-threatening/fatal), ^+^: classified as a TEAE only if meeting criteria for a serious TEAE

None of the four deaths reported during the study were considered treatment-related by the investigator (three in the BGF MDI group resulted from cerebral infarction, respiratory fume inhalation disorder, and sepsis; one in the GFF MDI group resulted from myocardial ischemia). Six patients (*n* = 3, BGF MDI; *n* = 3, GFF MDI) had events confirmed as MACE by the Clinical Endpoint Committee (four non-fatal myocardial infarctions and two cardiovascular deaths). The incidence of confirmed pneumonia was low overall (*n* = 11; 2.4%) and was highest in the GFF MDI group (3.4%), followed by BGF MDI (2.1%), and BFF MDI (1.1%; Table 6).

### Efficacy endpoints

**Fig. 2:**
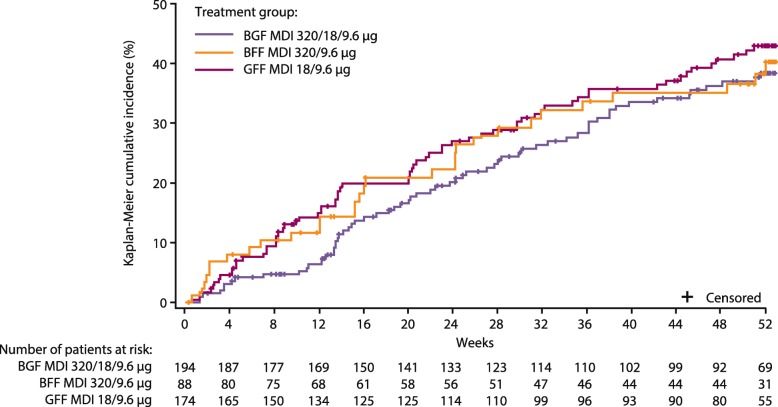
Kaplan-Meier curve for time to first moderate/severe COPD exacerbation (modified intent-to-treat population). *BFF* Budesonide/formoterol fumarate, *BGF* Budesonide/glycopyrrolate/formoterol fumarate, *COPD* Chronic obstructive pulmonary disease, *GFF* Glycopyrrolate/formoterol fumarate, *MDI* Metered dose inhaler

BGF MDI increased time to first moderate/severe COPD exacerbation over the entire treatment period relative to GFF MDI and throughout the majority of the treatment period relative to BFF MDI (Fig. 2). The rate of moderate/severe exacerbations over 52 weeks was lowest in the BGF MDI group (0.59), followed by BFF MDI (0.72), and GFF MDI (0.81; Additional file 1: Table S3). Reductions from baseline in daily rescue salbutamol use, percentage of rescue-free days and changes from baseline in EXACT scores were comparable across treatments (Additional file 1: Table S3)..

## Discussion

This study evaluated the long-term safety and tolerability of BGF MDI, BFF MDI, and GFF MDI in patients with moderate-to-very severe COPD over 52 weeks (i.e.*,* over the 24-week KRONOS study [2] and a 28-week extension). Because long-term use of ICS can be associated with increased risk of fractures and cataracts [16–19], BMD and ophthalmological assessments were selected as primary safety endpoints. To the best of our knowledge, this is the first study to specifically assess bone and ocular safety outcomes in patients with COPD following treatment with an ICS/LAMA/LABA triple FDC therapy.

Overall, changes in BMD and ophthalmological assessments were small in this study, and BGF MDI was non-inferior to the non-ICS-containing treatment (GFF MDI) for both primary endpoints. The effects of BGF MDI on BMD and ophthalmological assessments were similar in magnitude to those previously reported for budesonide/formoterol MDI in patients with moderate-to-very severe COPD [11]. Our study did not show evidence of any clinically meaningful ICS-induced changes in BMD or LOCS III scores. For instance, shifts in BMD T-scores from normal to osteopenic levels (>−2.5 and ≤−1) were similar in the BGF MDI and GFF MDI groups. Few patients across treatment groups experienced a worsening T-score for lumbar spine BMD relative to baseline, and no patients shifted to a lumbar spine T-score ≤−2.5 (indicative of osteoporosis) after 52 weeks of treatment. Although five patients (three receiving BGF MDI and one each in the BFF MDI and GFF MDI groups) had a total hip T-score ≤−2.5 at Week 52, each of these patients had a baseline T-score <−2, suggesting that this did not represent a major decline in BMD over the study period.

For LOCS III scores, no consistent changes attributable to ICS were seen in this study, and the incidence of Class 3 shifts was low (≤8.2%). While Class 1 and 2 increases were more frequent overall, Class 3 shifts are the most likely to represent a clinically meaningful treatment effect, rather than a result of aging or inter-assessment variability [21]. LOCS III shifts have been observed in patients with COPD receiving no active treatments, with a previous study finding that 18.6, 6.0, and 2.2% of patients receiving a placebo had Class 1, 2, and 3 increases, respectively, in P, NO, and/or C scores after 26 weeks [21]. Since our study did not have a placebo arm, we are unable to assess age-related deterioration in BMD and LOCS III scores, and many of the changes seen in BMD and ocular endpoints may be attributable to normal aging and COPD-related changes, rather than to any treatment component of BGF MDI.

The overall incidence of bone-related and ocular AEs was low and similar across treatment groups, consistent with other large randomized controlled trials in COPD that found no increase in rates of bone-related or ocular AEs in ICS treatment groups [14, 22].

Notably, after 52 weeks of treatment, pneumonia incidence was similar across groups, and slightly lower in the BGF MDI group relative to GFF MDI, confirming the findings of KRONOS [2] that the addition of budesonide to GFF MDI did not result in an apparent increased risk of pneumonia. In addition, there were no cumulative adverse effects of treatment over time as the incidence and types of TEAEs, including pneumonia, were generally similar in the first 24 weeks of the study and after Week 24. Overall, all treatments were well tolerated with no new or unexpected safety findings, and the safety profile of BGF MDI was similar to the well-characterized safety profiles of GFF MDI [23–25] and approved budesonide/formoterol combinations [11, 26].

In addition to the primary and secondary safety objectives of the study, several efficacy endpoints were also assessed. Although no inferential analyses were planned or performed, the finding that BGF MDI reduced the rate of moderate/severe exacerbations compared to GFF MDI and BFF MDI is in alignment with results from the 24-week KRONOS study [2], as well as previous studies comparing triple and dual FDC therapies over 52 weeks [3, 27, 28]. While all three treatments reduced rescue medication use and EXACT scores over 52 weeks, these findings should be interpreted in the context of the smaller sample size relative to the full KRONOS population.

One limitation of our study was that it excluded patients who had a baseline T-score <−2.5, which may have led to the exclusion of patients with the greatest risk profile for developing adverse events related to osteoporosis. However, the probability of detecting a change or decline in BMD in such patients would likely be limited. Strengths included the one-year study duration, the use of corresponding dual and triple therapies, assessments by LOCS III-certified ocular clinicians, and an adjudication committee to assess cardiovascular MACE events and pneumonia AEs using standardized criteria.

## Conclusions

Overall, this study found no clinically meaningful differences in BMD or ophthalmological safety assessments over 52 weeks after treatment with ICS-containing therapies BGF MDI and BFF MDI compared to the LAMA/LABA GFF MDI. All three treatments were well tolerated over 52 weeks with no new or unexpected safety findings, and the incidence of confirmed pneumonia was low and similar between BGF MDI and GFF MDI. In addition, there was no evidence of cumulative AEs, based on the incidence and types of AEs occurring in the first 24 weeks compared to after Week 24. The benefit of BGF MDI versus dual therapies on exacerbations was maintained when treatment was continued for up to 52 weeks. These findings provide additional evidence for the long-term tolerability of BGF MDI in patients with moderate-to-very severe COPD.

## Additional file

Additional file 1: Supplementary methods. **Table S1.** Important changes to methods after trial commencement. **Table S2.** TEAEs by time period (extension study safety population). **Table S3.** Efficacy endpoints (over 52 weeks; modified intent-to-treat population). Additional file **Figure S1.** Study design. Additional file **Figure S2.** Percent change from baseline in BMD of the lumbar spine (L2–L4) and total hip at Week 52 (BMD population). Additional file **Figure S3.** Change from baseline in LOCS III scores at Week 52 (ophthalmological population). (DOCX 325 kb)

## Data Availability

The datasets used and analyzed during the current study are available on reasonable request in accordance with AstraZeneca’s data sharing policy described at https://astrazenecagrouptrials.pharmacm.com/ST/Submission/Disclosure

## Abbreviations

AE: Adverse event
BFF: Budesonide/formoterol fumarate
BGF: Budesonide/glycopyrrolate/formoterol fumarate
BMD: Bone mineral density
C: Cortical cataract
CAT: COPD Assessment Test
CI: Confidence interval
COPD: Chronic obstructive pulmonary disease
DPI: Dry powder inhaler
EXACT: Exacerbations of Chronic Pulmonary Disease Tool
EXACT-RS: EXACT-Respiratory Symptom
FDC: Fixed-dose combination
FEV_1_: Forced expiratory volume in 1 s
GFF: Glycopyrrolate/formoterol fumarate
ICS: Inhaled corticosteroid
IOP: Intraocular pressure
LABA: Long-acting β_2_-agonist
LAMA: Long-acting anti-muscarinic antagonist
LOCS III: Lens opacities classification system III
LogMAR: Logarithm of the minimum angle of resolution
LSM: Least squares mean
MACE: Major adverse cardiovascular event
MDI: Metered dose inhaler
mITT: Modified intent-to-treat
NA: Not applicable
NC: Nuclear color
NO: Nuclear opalescence
P: Posterior subcapsular cataract
SD: Standard deviation
