# Supplementary material for: Bone and ocular safety of budesonide/glycopyrrolate/formoterol fumarate metered dose inhaler in COPD: a 52-week randomized study
Source: Respir Res. 2019 Jul 29;20:167. doi: 10.1186/s12931-019-1126-7 (PMC6664772; doi:10.1186/s12931-019-1126-7)
Supplement: Supplementary file 1 — Supplementary methods. Table S1. Important changes to methods after trial commencement. Table S2. TEAEs by time period (extension study safety population). Table S3. Efficacy endpoints (over 52 weeks; modified intent-to-treat population). Additional file Figure S1. Study design. Additional file Figure S2. Percent change from baseline in BMD of the lumbar spine (L2–L4) and total hip at Week 52 (BMD population). Additional file Figure S3. Change from baseline in LOCS III scores at Week 52 (ophthalmological population). (DOCX 325 kb) [file 12931_2019_1126_MOESM1_ESM.docx]

**Additional file 1**

# Supplementary methods

## Bone mineral density assessments

Bone mineral density (BMD) scans of each skeletal site were centrally analyzed by BioClinica Inc. (Newtown, PA, USA) to obtain the final result used for data analysis. Detailed procedure manuals were provided for both Hologic and GE Lunar scanner models to standardize the dual-energy X-ray absorptiometry scanning procedures across participating investigative sites. To ensure consistency, the appropriate scan acquisition mode for each scanner model was specified in the procedure manual. Calibration with a phantom was performed at baseline and prior to the first patient measurement at Week 52. BMD results were provided by the central analysis facility to the investigative site to allow appropriate management of the patient.

## Ophthalmological assessments

At participating centers, patients were referred to a certified Lens Opacities Classification System III (LOCS III)-trained ophthalmologist/optometrist for evaluation of pupil dilation, lenticular opacities, fundoscopic examination, intraocular pressure (IOP), manifest refraction and visual acuity. To ensure consistency, the same ophthalmologist performed the ophthalmic examinations for a given patient at all visits if possible. LOCS III assessments were performed using a decimalized scale ranging from 0.1 (indicating a completely clear or colorless lens) to either 5.9 (indicating complete opacification on the cortex or posterior capsule for cortical cataract [C] or severity of posterior subcapsular cataract [P] scales) or 6.9 (indicating advanced opacification and brunescence of the nucleus for nuclear opalescence [NO] or nuclear color [NC] scales) [1]. Each assessment was recorded to the nearest 0.1.

IOP was measured using Goldmann applanation tonometry; three measurements were completed and recorded for each eye during each ophthalmic examination. Visual acuity (best-corrected distance vision) and manifest refraction (sphere, cylinder, and axis) were assessed during each ophthalmic examination. Logarithm of the minimum angle of resolution (LogMAR) visual acuity scores were assessed using Early Treatment Diabetic Retinopathy Study charts.

## Additional safety assessments

Adverse events of special interest included cardiovascular effects, ocular disorders, urinary retention, gastrointestinal disorders and anticholinergic effects for long-acting anti-muscarinic antagonists; cardiovascular, tremor effects, hyperglycemia and hypokalemia for long-acting β_2_-agonists; and local (e.g. candidiasis and voice effects) and systemic (e.g. bone and skin effects, diabetes control, ocular and taste effects, adrenal suppression) steroid class effects and lung infection for inhaled corticosteroids.

## Statistical analysis

A sample size of 500 randomized patients from KRONOS was selected to provide 425 patients with baseline and on-treatment BMD and LOCS III assessments, assuming a 15% rate of discontinuation through Week 52. This sample size would provide ~97% power to demonstrate non-inferiority of budesonide/glycopyrrolate/formoterol fumarate (BGF) metered dose inhaler (MDI) to glycopyrrolate/formoterol fumarate (GFF) MDI in the primary BMD endpoint (~91% power for budesonide/formoterol fumarate (BFF) MDI *versus* GFF MDI), based on a lower 95% confidence interval limit of −2%, a true difference of −0.3%, and standard deviation of 4%. For the primary ophthalmological endpoint, it would provide >99% power to demonstrate non-inferiority of BGF MDI to GFF MDI (or BFF MDI to GFF MDI) based on an upper 95% confidence interval limit of 0.5 (1-sided, alpha=0.025) assuming no true difference in means.

The percentage change from baseline in BMD of the lumbar spine and total hip at Week 52 was analyzed using an analysis of covariance model which included treatment group and gender as categorical covariates, and baseline natural-log-transformed BMD score, age, and age by gender interaction as continuous covariates. Changes from baseline in LOCS III (P, NO, NC and C) score and IOP were analyzed using a repeated measures linear mixed model, adjusting for treatment, visit and the treatment by visit interaction as categorical covariates; and baseline value, smoking pack-years, and age as continuous covariates. Eye (within patient) was included as a random effect. The IOP was measured three times per eye, and the analysis value was calculated as the median of the IOP measurements for a given eye. Changes from baseline in LogMAR visual acuity scores and horizontal cup-to-disc ratio were analyzed across eyes (irrespective of patient [each eye that was measured was given equal weight; *i.e.,* if a patient had only one eye assessed, that eye was not given twice the weight]) at each visit by study drug using descriptive statistics.

# Supplementary results

## Study population

Seventy-two patients who consented to participate in the extension study when they entered KRONOS discontinued treatment prior to Week 24. Of these, 18 patients discontinued due to the following AEs (some patients discontinued due to multiple AEs): muscle spasms (n=2), acute pyelonephritis/acute kidney injury/sepsis/urinary tract infection, acute respiratory failure/pneumonia, alcohol abuse, asthenia, atrial fibrillation, atrial flutter, cerebral infarction, chest discomfort/oropharyngeal pain, congestive cardiac failure, dyspnea, electrocardiogram T wave inversion, metastases to lung, oral candidiasis, pulmonary mass, respiratory fume inhalation disorder, and sepsis.

# Reference

1. Chylack LT, Wolfe JK, Singer DM, Leske C, Bullimore MA, Bailey IL, Friend J, McCarthy D, Wu SY. The Lens Opacities Classification System III. Arch Ophthalmol. 1993:111:831–836.

**Table S1** Important changes to methods after trial commencement

| **Description of change** | **Rationale** |
| --- | --- |
| Revised BMD exclusion criteria:   - *To apply to either hip or lumbar region*  Added additional BMD exclusion criteria:*Subjects unable to achieve an acceptable scan (e.g., due to the patient’s inability to be stable during the procedure, due to the limitation of scanning equipment to accommodate a subject (~300 lbs or ~136 kgs) or other characteristics)* | Clarified to indicate that criteria applied to either the lumbar or the hip region and added exclusion criteria to account for limitations of the scanning equipment |
| Revised ophthalmological criteria:   - *To apply to either eye* - *IOP >21 mmHg (lowest of the 3 readings)* | Clarified to indicate that these criteria applied to either eye and revised to indicate that the lowest of the 3 IOP readings should be considered to evaluate the exclusion of IOP >21 mmHg |
| Added exclusion criteria exception:   - *Subjects who meet none of BMD exclusion criteria, but who meet one or more ophthalmological exclusion criteria, may be permitted to continue in the study. Such subjects would undergo all study planned assessments except they would undergo no further ophthalmological exams during the randomized treatment period. Additionally, such subjects would be permitted to continue in the study provided that the following 2 conditions are satisfied:*  *IOP is ≤ 22 mmHg either with or without treatment*  - - *In the opinion of the optometrist/ophthalmologist, the subject does not have a condition (e.g., advanced glaucoma) that the eye health of the subject could be jeopardized by continued participation in the study* | Added to allow subjects who meet none of the BMD exclusion criteria, but one or more of the ophthalmological exclusion criteria, to continue in the study based on the specified conditions |
| Extended BMD screening period:   - *The screening period may be extended up to a maximum of 21 days if additional time is needed to complete the assessments* | Extended to allow adequate time to complete assessments during the screening period |
| BMD: bone mineral density; IOP: intraocular pressure. | |

**Table S2** TEAEs by time period (extension study safety population)

|  | **BGF MDI**  **320/18/9.6 µg**  **(*N* = 160)** | **BFF MDI**  **320/9.6 µg**  **(*N* = 70)** | **GFF MDI**  **18/9.6 µg**  **(*N* = 148)** | **All**  **patients**  **(*N* = 378)** |
| --- | --- | --- | --- | --- |
| **TEAEs (0 to ≤24 weeks)** | | | | |
| Patients with ≤1 TEAE | 102 (63.8) | 40 (57.1) | 89 (60.1) | 231 (61.1) |
| Patients with treatment-related TEAEs^#^ | 19 (11.9) | 11 (15.7) | 14 (9.5) | 44 (11.6) |
| Patients with serious TEAEs | 11 (6.9) | 2 (2.9) | 9 (6.1) | 22 (5.8) |
| Patients with treatment-related serious TEAEs^#^ | 0 | 0 | 0 | 0 |
| Patients with confirmed MACE^¶^ | 0 | 0 | 1 (0.7) | 1 (0.3) |
| Patients with confirmed pneumonia^¶^ | 2 (1.3) | 1 (1.4) | 3 (2.0) | 6 (1.6) |
| Deaths (all causes) | 0 | 0 | 0 | 0 |
| TEAEs occurring in ≥4% of patients in any treatment arm, n (%) |  |  |  |  |
| Upper respiratory tract infection  Nasopharyngitis  Muscle spasms  Hypertension  Dysphonia | 9 (5.6)  6 (3.8)  1 (0.6)  4 (2.5)  2 (1.3) | 3 (4.3)  4 (5.7)  7 (10.0)  3 (4.3)  4 (5.7) | 7 (4.7)  5 (3.4)  4 (2.7)  3 (2.0)  2 (1.4) | 19 (5.0)  15 (4.0)  12 (3.2)  10 (2.6)  8 (2.1) |
| **TEAEs (>24 to ≤52 weeks)** |  |  |  |  |
| Patients with ≤1 TEAE | 88 (55.0) | 36 (51.4) | 86 (58.1) | 210 (55.6) |
| Patients with treatment-related TEAEs^#^ | 9 (5.6) | 7 (10.0) | 13 (8.8) | 29 (7.7) |
| Patients with serious TEAEs | 18 (11.3) | 3 (4.3) | 11 (7.4) | 32 (8.5) |
| Patients with treatment-related serious TEAEs^#^ | 1 (0.6) | 0 | 1 (0.7) | 2 (0.5) |
| Patients with confirmed MACE^¶^ | 2 (1.3) | 0 | 2 (1.4) | 4 (1.1) |
| Patients with confirmed pneumonia^¶^ | 2 (1.3) | 0 | 2 (1.4) | 4 (1.1) |
| Deaths (all causes) | 0 | 0 | 1 (0.7) | 1 (0.3) |
| TEAEs occurring in ≥4% of patients in any treatment arm, n (%) |  |  |  |  |
| Upper respiratory tract infection  Viral upper respiratory tract infection  COPD  Sinusitis  Urinary tract infection  Bronchitis | 9 (5.6)  8 (5.0)  7 (4.4)  7 (4.4)  6 (3.8)  3 (1.9) | 3 (4*.*3)  4 (5.7)  1 (1.4)  1 (1.4)  3 (4.3)  0 | 12 (8.1)  5 (3.4)  4 (2.7)  3 (2.0)  1 (0.7)  6 (4.1) | 24 (6.3)  17 (4.5)  12 (3.2)  11 (2.9)  10 (2.6)  9 (2.4) |
| Data are n (%). BFF: budesonide/formoterol fumarate; BGF: budesonide/glycopyrrolate‌/formoterol fumarate; COPD: chronic obstructive pulmonary disease; GFF: glycopyrrolate‌/formoterol fumarate; MACE: major adverse cardiovascular event; MDI: metered dose inhaler; TEAE: treatment-emergent adverse event. ^#^: possibly, probably, or definitely related in the opinion of the investigator; **^¶^**: confirmed by a clinical endpoint committee. | | | | |

**Table S3** Efficacy endpoints (over 52 weeks; modified intent-to-treat population)

|  | **BGF MDI 320/18/9.6 µg (*N* = 194)** | **BFF MDI 320/9.6 µg (*N* = 88)** | **GFF MDI 18/9.6 µg (*N* = 174)** |
| --- | --- | --- | --- |
| **Rescue medication use** | | | |
| Change from baseline in mean daily number of puffs of rescue salbutamol, mean (SD) | −0.5 (1.7)  *n*= 194 | −0.8 (2.0)  *n*= 86 | −0.3 (2.0)  *n*= 174 |
| Percentage of days with no rescue salbutamol use, mean (SE) | 53.2 (2.8)  *n*= 194 | 54.2 (4.4)  *n*= 87 | 50.0 (3.1)  *n*= 174 |
| **Exacerbations** |  |  |  |
| Patients with moderate/severe COPD exacerbations, n (%) | 64 (33.0) | 30 (34.1) | 67 (38.5) |
| Rate of moderate/severe COPD exacerbations per year^#^, n (%) | 0.59 | 0.72 | 0.81 |
| **Change from baseline in mean daily EXACT scores, mean (SD)** | | | |
| n | 194 | 86 | 174 |
| EXACT total score | −2.1 (6.4) | −2.0 (6.7) | −1.8 (5.2) |
| RS-total score | −1.0 (3.1) | −1.0 (3.4) | −0.7 (3.1) |
| RS-breathlessness score | −0.4 (1.8) | −0.4 (1.8) | −0.4 (1.6) |
| RS-cough and sputum score | −0.2 (0.9) | −0.3 (1.0) | −0.2 (1.0) |
| RS-chest symptoms score | −0.3 (1.1) | −0.3 (1.1) | −0.1 (1.2) |
| BFF: budesonide/formoterol fumarate; BGF: budesonide/glycopyrrolate/formoterol fumarate; EXACT: Exacerbations of Chronic Pulmonary Disease Tool; GFF: glycopyrrolate/formoterol fumarate; MDI: metered dose inhaler; RS: respiratory symptom; SD: standard deviation; SE: standard error. ^#^: rate of exacerbations per year = total number of exacerbations/total years of exposure across all patients for the treatment. | | | |

**Fig. S1** Study design


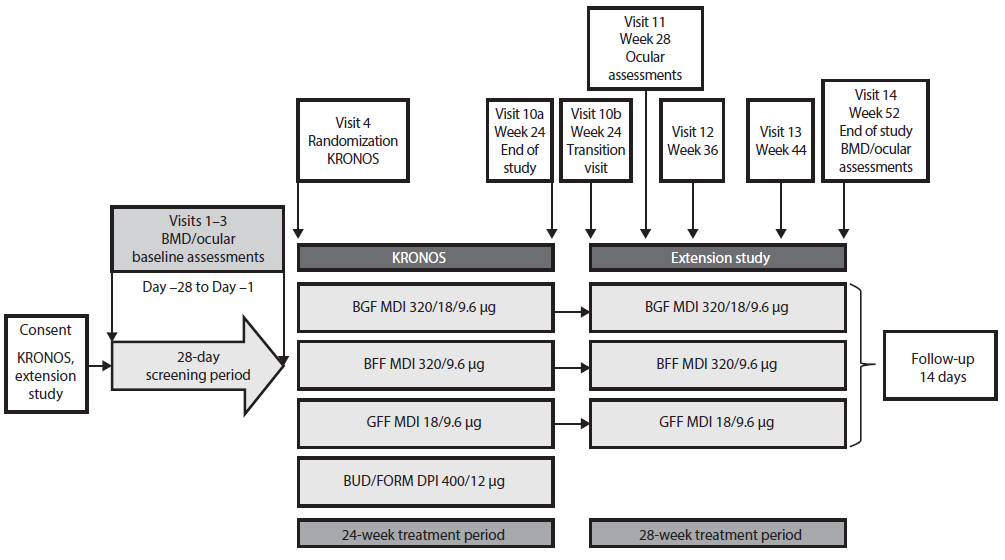


BFF: budesonide/formoterol fumarate; BGF: budesonide/glycopyrrolate/formoterol fumarate; BMD: bone mineral density; BUD/FORM: budesonide/formoterol fumarate; DPI: dry powder inhaler; GFF: glycopyrrolate/formoterol fumarate; MDI: metered dose inhaler.

**Fig. S2** Percent change from baseline in BMD at Week 52 (BMD population)


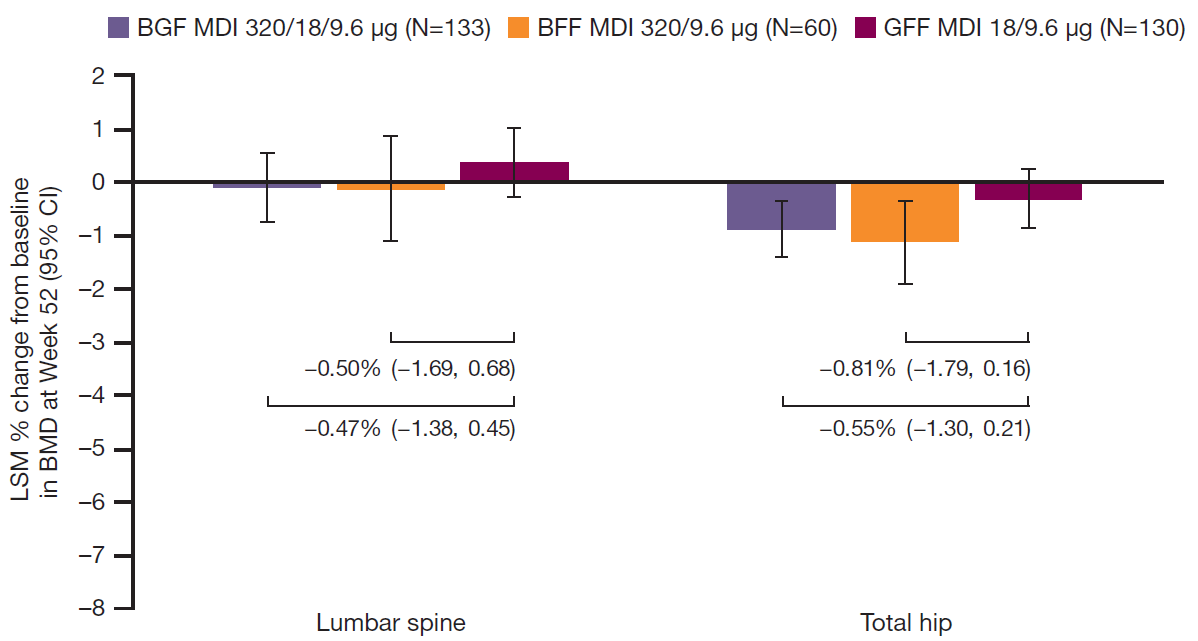


Horizontal bars between treatments are labeled with LSM difference (95% CI).

BFF: budesonide/formoterol fumarate; BGF: budesonide/glycopyrrolate/formoterol fumarate; BMD: bone mineral density; CI: confidence interval; GFF: glycopyrrolate/formoterol fumarate; L: lumbar spine segment; LSM: least squares mean; MDI: metered dose inhaler.

**Fig. S3** Change from baseline in LOCS III scores at Week 52 (ophthalmological population)


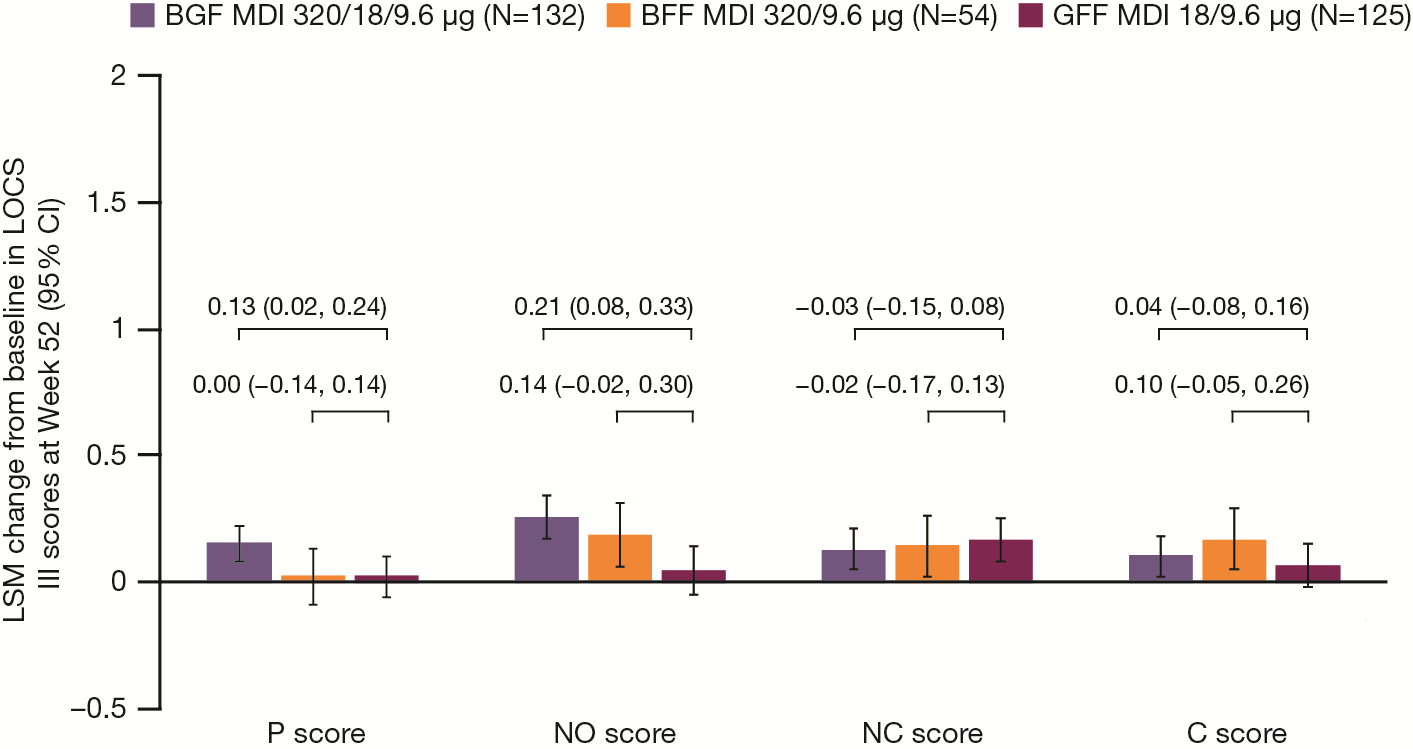


Horizontal bars between treatments are labeled with LSM difference (95% CI).

BFF: budesonide/formoterol fumarate; BGF: budesonide/glycopyrrolate/formoterol fumarate; C: cortical cataract; CI: confidence interval; GFF: glycopyrrolate/formoterol fumarate; LOCS III: lens opacities classification system III; LSM: least squares mean; MDI: metered dose inhaler; NC: nuclear color; NO: nuclear opalescence; P: posterior subcapsular cataract.
